# Inflammation-Induced Alternative Pre-mRNA Splicing in Mouse Alveolar Macrophages

**DOI:** 10.1534/g3.119.400935

**Published:** 2019-12-06

**Authors:** William J. Janssen, Thomas Danhorn, Chelsea Harris, Kara J. Mould, Frank Fang-Yao Lee, Brenna R. Hedin, Angelo D’Alessandro, Sonia M. Leach, Scott Alper

**Affiliations:** * Department of Medicine,; ‡ Center for Genes, Environment and Health, and; § Department of Biomedical Research, National Jewish Health, Denver, CO, 80206,; ** Department of Immunology and Microbiology, University of Colorado School of Medicine, Aurora, CO, 80045,; † Division of Pulmonary Sciences and Critical Care Medicine, and; †† Department of Biochemistry and Molecular Genetics, University of Colorado Denver, Aurora, CO, 80045

**Keywords:** alternative pre-mRNA splicing, alveolar macrophage, lipopolysaccharide, glycolysis, tricarboxylic acid cycle

## Abstract

Alveolar macrophages serve as central orchestrators of inflammatory responses in the lungs, both initiating their onset and promoting their resolution. However, the mechanisms that program macrophages for these dynamic responses are not fully understood. Over 95% of all mammalian genes undergo alternative pre-mRNA splicing. While alternative splicing has been shown to regulate inflammatory responses in macrophages *in vitro*, it has not been investigated on a genome-wide scale *in vivo*. Here we used RNAseq to investigate alternative pre-mRNA splicing in alveolar macrophages isolated from lipopolysaccharide (LPS)-treated mice during the peak of inflammation and during its resolution. We found that lung inflammation induced substantial alternative pre-mRNA splicing in alveolar macrophages. The number of changes in isoform usage was greatest at the peak of inflammation and involved multiple classes of alternative pre-mRNA splicing events. Comparative pathway analysis of inflammation-induced changes in alternative pre-mRNA splicing and differential gene expression revealed overlap of pathways enriched for immune responses such as chemokine signaling and cellular metabolism. Moreover, alternative pre-mRNA splicing of genes in metabolic pathways differed in tissue resident *vs.* recruited (blood monocyte-derived) alveolar macrophages and corresponded to changes in core metabolism, including a switch to Warburg-like metabolism in recruited macrophages with increased glycolysis and decreased flux through the tricarboxylic acid cycle.

Acute inflammation is necessary to combat infection but also causes tissue damage. Thus, proper resolution of inflammation is critical for organ recovery once an infection is cleared. Alveolar macrophages (AMs) reside in the airspaces and airways where they constantly survey the environment and maintain homeostasis by engulfing inhaled particulates and debris. In addition, they serve as central orchestrators of the inflammatory response, both regulating its onset and its resolution. In order to serve these divergent and seemingly opposing functions, AMs undergo dynamic, stimulus-specific programming. While the factors that regulate this programming are not fully understood, it is clear that changes in gene expression and cellular metabolism play critical roles. For instance, when a macrophage first encounters a bacterial pathogen, specialized pathogen recognition receptors (PRRs) initiate complex transcriptional responses that promote the production of inflammatory cytokines, chemokines and other host defense molecules (Medzhitov and Horng 2009; Takeuchi and Akira 2010). At the same time, core metabolism is switched from oxidative phosphorylation to glycolysis (Jha *et al.* 2015; Rodríguez-Prados *et al.* 2010). However, once pathogens are cleared, macrophages express genes involved in the resolution of inflammation (Hamidzadeh *et al.* 2017). Meanwhile, metabolic flux through the tricarboxylic acid (TCA) cycle is restored. The factors that regulate these transitions are complex; we hypothesize that these transitions depend, in part, on alternative pre-mRNA splicing.

Alternative pre-mRNA splicing is a highly regulated process that enables single genes to generate multiple distinct mRNAs that encode distinct proteins. It is estimated that ∼95% of all multi-exon human genes undergo alternative splicing (Lee and Rio 2015). Thus alternative pre-mRNA splicing greatly enhances the complexity of the proteome (Lee and Rio 2015). Much of this occurs in a cell-type-specific and/or signal-induced manner. We and others, have shown that mouse and human macrophages exposed to inflammatory stimuli *in vitro* undergo substantial alternative pre-mRNA splicing (Beyer *et al.* 2012; Bhatt *et al.* 2012; de Bruin *et al.* 2016; Haque *et al.* 2018; Lin *et al.* 2016; Liu *et al.* 2018; O’Connor *et al.* 2015; Pai *et al.* 2016; Pandya-Jones *et al.* 2013). This can have profound effects on the nature and extent of the inflammatory response (Lynch 2004; Schaub and Glasmacher 2017). For example, alternative pre-mRNA splicing can result in production of inflammatory signaling molecules with altered activity or stability (Cadalbert *et al.* 2010; Han *et al.* 2010; Phan *et al.* 2006; Wells *et al.* 2006). Additionally, some genes that encode positive effectors of inflammatory signaling can also produce alternate pre-mRNA splice forms that encode negative regulators of signaling (Blumhagen *et al.* 2017; De Arras and Alper 2013; Deng *et al.* 2008; Gray *et al.* 2010; Hardy and O’Neill 2004; Iwami *et al.* 2000; Janssens *et al.* 2002; Koop *et al.* 2011; Palsson-McDermott *et al.* 2009; Rao *et al.* 2005; Rosenstiel *et al.* 2006), thus mediating a negative feedback loop to limit the extent of the inflammatory response. In a similar fashion, alternative pre-mRNA splicing has been shown to alter cellular metabolism (Clower *et al.* 2010; Yang and Lu 2013; Satoh *et al.* 2015).

While inflammation-induced alternative pre-mRNA splicing in macrophages has been investigated on a genome-wide scale *in vitro* (Beyer *et al.* 2012; Bhatt *et al.* 2012; Lin *et al.* 2016; O’Connor *et al.* 2015; Pai *et al.* 2016; Pandya-Jones *et al.* 2013), to our knowledge it has not been investigated *in vivo*. Such an approach is necessary to fully appreciate the effect of *in vivo* physiological context on macrophage pre-mRNA splicing. In the current study, we examined alternative pre-mRNA splicing on a genome-wide scale in murine alveolar macrophage (AM) subsets isolated at selected points after LPS-induced inflammation.

In line with our previous studies (Janssen *et al.* 2011; Mould *et al.* 2017; Mould *et al.* 2019), two unique AM subsets were assessed. These included *residen*t AMs that reside in the airspaces during health and persist throughout inflammation, and *recruited* AMs that arise from circulating blood monocytes that migrate to the lungs during early inflammation (Janssen *et al.* 2011; Mould *et al.* 2017; Mould *et al.* 2019). Resident AMs serve as sentinels that continuously survey the airways and alveoli. When resident AMs first encounter a pathogen, innate immune signaling pathways induce the release of pro-inflammatory cytokines, chemokines, and other host defense molecules (Aggarwal *et al.* 2014; Huang *et al.* 2018). These promote rapid recruitment of neutrophils and monocytes to sites of infection. Monocytes that subsequently mature into recruited AMs contribute to the inflammatory response and promote further tissue damage. As inflammation resolves, recruited AMs become reprogrammed for tissue reparative functions (Aggarwal *et al.* 2014; Huang *et al.* 2018; Watanabe *et al.* 2019). Once tissues are repaired, recruited AMs undergo apoptosis, and resident AMs are left to serve as homeostatic sentinels (Janssen *et al.* 2011).

We previously demonstrated that inflammation induces distinct transcriptional programs in the two AM populations, and that this response varies over the course of inflammation (Mould *et al.* 2017). In the current investigation, we took advantage of this prior study to investigate inflammation-induced alternative pre-mRNA splicing at the genomic scale in parallel with targeted metabolomics in resident and recruited AMs. We find that: (1) inflammation induces substantial alternative pre-mRNA splicing in both resident and recruited AMs, (2) alternative splicing occurs primarily during the early pro-inflammatory phase of inflammation and not during its resolution, and (3) metabolic and inflammatory signaling pathways targeted by alternative splicing overlap substantially with pathways enriched for differentially expressed genes, suggesting that these alternate splicing events play an important role in modulating the biological response to inflammation. We also find that resident and recruited AM populations differ significantly in their isoform usage; in particular, we find significant alternative isoform usage in genes that govern cellular metabolism. Thus, there are both inflammation-induced and cell-specific differences in pre-mRNA splicing in AMs.

## Materials and Methods

### Mouse studies and analysis of LPS-induced inflammatory cytokine production

Mouse studies were approved by the National Jewish Health Institutional Animal Care and Use Committee (IACUC). Experiments were performed on C57BL/6J mice 10-12 weeks of age (Jackson Laboratories, Bar Harbor, ME). Mice were anesthetized with isoflurane, and 20 µg of *E. coli* O55:B5 LPS (List Biological Laboratories, Campbell, CA) dissolved in 50 µl of PBS was instilled directly into mouse tracheas using a modified feeding needle. Mice were euthanized with intraperitoneal Fatal Plus in accordance with American Veterinary Medical Association guidelines. Tracheas were identified with blunt dissection and an 18g needle was inserted. Bronchoalveolar lavage was performed using PBS supplemented with 5 mM EDTA. 5 × 1 ml lavages were performed. Fluid from the first lavage aliquot was centrifuged to pellet cells, and 0.5 ml of supernatant was withdrawn and used for cytokine analysis. Cells from the first aliquot were resuspended in 0.5 ml PBS and added to aliquots 2-5. Leukocytes were enumerated with a hemocytometer and cell differentials performed using light microscopy with Wright-Giemsa stained cytospins. Cytokine concentrations were measured on cell-free bronchoalveolar lavage fluid using the Milliplex mouse cytokine/chemokine magnetic bead kit (Millipore Sigma, Burlington, MA) following the manufacturer’s instructions.

### Isolation of resident and recruited alveolar macrophages (AMs)

**Figure 1:**
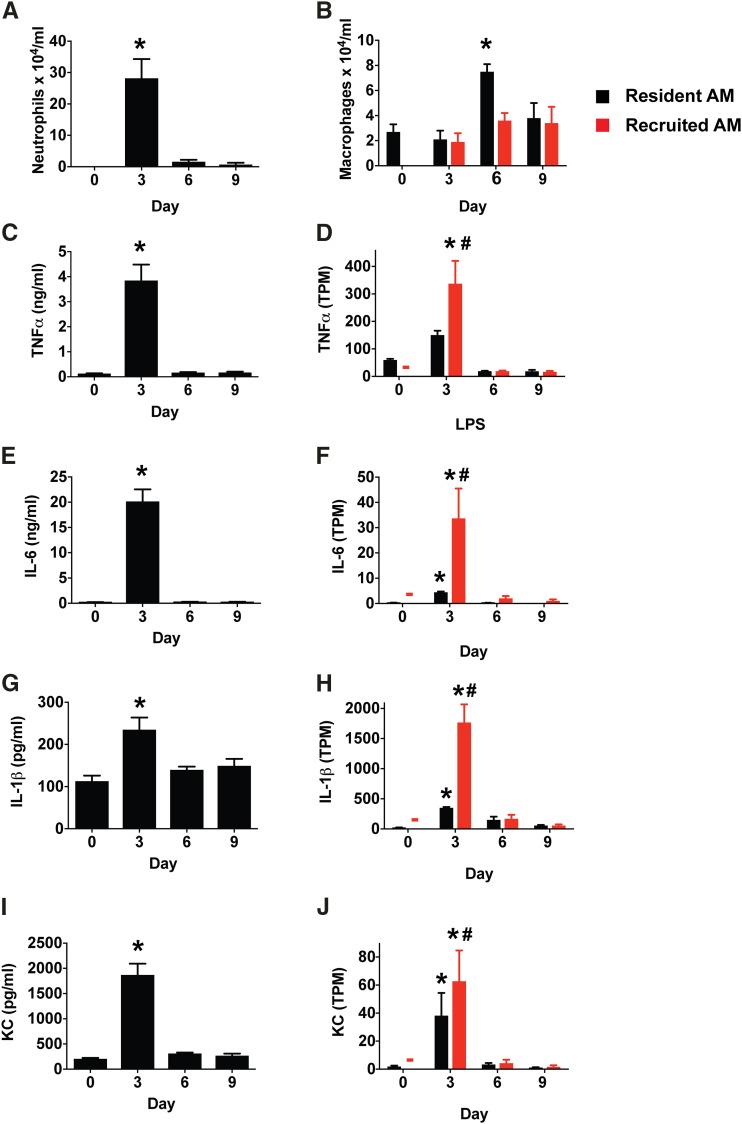
Intratracheal LPS administration induces transient production of pro-inflammatory cytokines. Mice were treated with intratracheal LPS (20 μg). Bronchoalveolar lavage fluid was collected 3, 6, and 9 days later. Day 0 indicates naive animals. (A, B) neutrophil and macrophage numbers in lavage fluid. N ≥ 6 mice per group. (C, E G, I) inflammatory cytokine proteins in bronchoalveolar lavage fluid assayed by ELISA. N = 4 mice per group. (D, F, H, J) gene expression (transcripts per million, TPM) of pro-inflammatory cytokines in resident and recruited alveolar macrophages (AM). N = 3 biologic replicates per time point. Data represent mean, SD. *P < 0.05 *vs.* other time points. #*P* < 0.05 *vs.* resident AM from LPS Day 3. The red dash is a reminder that there is no Day 0 sample for recruited AM.

Samples for RNAseq were obtained as described (Mould *et al.* 2017). Pooled bronchoalveolar lavage samples were washed twice in PBS and stained on ice for 30 min with antibodies directed at Ly6G, CD3, B220, NK1.1, CD64, CD11b, CD11c and F4/80. The following antibodies were used at the indicated concentrations: anti-Ly6G (BD Biosciences, Clone 1A8, 2.5 μg/ml), anti-CD3 (EBioscience Clone 17A2, 2.5 μg/ml), anti-B220 (Ebioscience, Clone RA3-6B2, 1 μg/ml), anti-NK1.1 (EBioscience, Clone PK136, 1 μg/ml), anti-CD64 (BD Pharmingen Clone X54-5/7.1, 1 μg/ml), anti-CD11c (Ebioscience, clone N418, 1 μg/ml), anti-CD11b (Ebioscience, clone M1/70, 1 μg/ml), anti-F4/80 (Ebioscience, clone BM8 1 μg/ml). Samples from day 3 were incubated with biotinylated anti-Ly6G antibody for 15 min, washed with PBS and incubated with magnetic anti-biotin microbeads. Neutrophils were depleted using magnetic columns (Miltenyi Biotech, BergischGladbach, Germany). Flow-through from the columns was collected and washed in PBS prior to staining for fluorescence activated cell sorting (FACS). DAPI (4′,6-diamidino-2-phenylindole from Biolegend, #422801) was added to cell suspensions immediately prior to FACs to label dead cells. Resident and recruited AMs were purified using FACS with a FACSAria Fusion (BD Biosciences, Franklin Lakes, NJ) using the following strategy. First, forward-scatter and side scatter were used to identify viable cells and eliminate debris. Doublets were then excluded using forward-scatter area and forward-scatter width. Next neutrophils, lymphocytes and dead cells (permeable to DAPI) were excluded. Macrophages were then identified by their high expression of F4/80 and CD64. Finally, resident AM were identified as CD11c^high^, CD11b^low^ cells, whereas recruited macrophages were CD11c^low^, CD11b^high^. A representative flow cytometry plot demonstrating the isolation and purity of these populations is available in Figure 1 in (Mould *et al.* 2017). Because recruited AMs are not present until after LPS challenge, there is no Day 0 recruited AM sample.

### Analysis of RNAseq data

Generation of the RNAseq data (Gene Expression Omnibus accession number GSE94749) and analysis of differential gene expression has been described previously (Mould *et al.* 2017). After library construction, the libraries were sequenced as bar-coded pooled samples on a P1 Ion Proton chip. We obtained between 15.1 and 25.2 million reads (19.7 million average) per sample; the read length averages ranged from 127.7 to 154.6 nucleotides (143.2 nucleotide average). Differential gene expression was detected using DESeq2 (Anders and Huber 2010) (version 1.4.5 under R version 3.1.0). In the current study, alternative isoform usage was detected using DEXSeq (version 1.10.8 under R version 3.1.0, using exon annotation from Ensembl version 71), which analyzes exon-by-exon expression level changes in RNAseq data (Anders *et al.* 2012). While this is expected to identify primarily exon skipping or alternative exon retention events, in our experience (O’Connor *et al.* 2015), this software also detects many intron retention events because of the myriad of isoforms annotated in Ensembl, some of which have exons in canonically intronic areas. Relevant sample combinations were further analyzed for alternative splicing using two additional software packages: MISO (version 0.4.9, using version 2 of the mouse mm10 splice event annotation provided at http://genes.mit.edu/burgelab/miso/annotations/ver2/), which uses predetermined alternative isoform models of adjacent exons to identify alternate pre-mRNA splicing events (Katz *et al.* 2010), and Cuffdiff (version 2.1.1), which identifies alternative pre-mRNA splicing using transcript information reconstructed from the sequence reads of all samples (Trapnell *et al.* 2013). Different software packages were used for different analyses. Pathway analysis was performed on the DEXseq data, because this package identified the largest set of alternative splicing events. MISO was used to quantify the nature of the different alternative splicing events, because MISO stratifies the data in this fashion. While some of the analysis methods often report more than one alternative splicing event per gene, we are only counting the affected genes, as this best reflects the biological effect and avoids confusion about which detected “events” are actually independent *vs.* part of the same splicing change.

The analysis of classes of alternate splicing events induced by LPS used the gene lists generated by MISO with a Bayes factor ≥10 cutoff.

### Pathway analysis

Pathway analysis was performed using DAVID 6.8 (Huang da *et al.* 2009) to identify differentially regulated KEGG pathways (Kanehisa and Goto 2000). Genes with alternative splicing events (adjusted *P* < 0.05) identified using DEXSeq were analyzed in DAVID, and pathways with *P* < 0.1 (after adjusting for multiple testing by the Benjamini method) were considered significant. Overlap among pathways was detected using Microsoft Excel; Venn diagrams displaying pathway overlap were generated using eulerr (Larsson 2019).

### qPCR and RT-PCR to monitor isoform-specific mRNA levels

qPCR was performed on an ABI QuantStudio7 Flex (Applied Biosystems, Foster City, CA) using the Quantitect SYBR-Green RT-PCR kit (Qiagen Sciences, Valencia, CA). This kit performs the reverse transcription reaction and subsequent PCR amplification using a single reaction mix. Data were analyzed using the ddCt method; isoform expression was normalized relative to βactin expression. qPCR was performed on three independent biological replicates. Differential gene expression was analyzed for significance using unpaired *t*-tests (Graphpad Prism 5, GraphPad Software, Inc., La Jolla, CA). Primer sets used for expression analysis are listed in Table S1. Wild type Nemo mRNA was monitored using a forward primer that annealed to exon 1 and a reverse primer that annealed to the exon 2-exon 1 junction. NemoΔ2 mRNA was monitored using a forward primer that annealed to exon 1 and a reverse primer that annealed to the unique exon 3-exon 1 junction. Cav1α mRNA was assayed using a forward primer that crossed the exon 1-exon 2 junction and a reverse primer that annealed to exon 2. Cav1β was assayed using a primer that crossed the novel junction in the first exon and the same reverse primer. Ogdh-4A (transcript NM_001252288.1) was analyzed using a forward primer that annealed to the exon 3-exon 4A junction and a reverse primer that annealed to exon 4A. Likewise, Ogdh-4B (transcript NM_001252287.1) was analyzed using a forward primer that annealed to the exon 3-exon 4B junction and a reverse primer that annealed to exon 4B. Primer efficiencies (Pfaffl 2001) for qPCR were calculated using synthesized isoform-specific DNA templates (sequences in Table S1) and are 1.76 for Ogdh-4A, 1.71 for Ogdh-4B, 1.77 for Cav1α, and 1.75 for Cav1β. Because the isoforms amplified with similar efficiencies, we used the qPCR results to directly compare isoform levels.

Nemo and NemoΔ2 mRNA were also assessed semi-quantitatively by reverse transcribing purified RNA using a gene-specific RT primer (Table S1) and the GoScript Reverse transcription system (Promega), PCR amplifying with primers that bracket exon 2 (Table S1) using the GoTaq PCR kit (Promgega), and subjecting the PCR products to agarose gel electrophoresis. The product corresponding to Nemo mRNA was 529 bp; the product corresponding to NemoΔ2 mRNA was 381 bp. Band intensities were quantitated using NIH imageJ (Schneider *et al.* 2012), normalized relative to band size, and estimates of the two isoform levels were performed.

### Metabolomics

For isotope-labeling experiments, resident and recruited AMs were isolated on Day 3 after LPS treatment as described above, except that mice were injected via the tail vein with 100 microliters of 5% U-^13^C-glucose (Cambridge Isotope Laboratories, Inc, Tewksbury, MA) in PBS at 30 and 60 min prior to euthanasia. Frozen cell pellets were extracted in ice cold lysis/extraction buffer (methanol:acetonitrile:water 5:3:2) at 1x10^6^ cells per milliliter. Samples were processed and metabolomics performed as previously reported (Mould *et al.* 2017). Metabolite assignments and isotopologue distributions (upon correction for natural abundances) were determined using Maven (Princeton, NJ) following conversion of .raw files to .mzXML format using MassMatrix (Cleveland, OH). Technical stability was assessed by determining coefficients of varitation for technical mixtures run every three injections. Statistical analyses of metabolomics data were performed through the software GENE E (Broad Institute, Boston, MA) and Metaboanalyst 3.0.

### Data availability

The authors affirm that all data necessary for confirming the conclusions of the article are present within the article, figures, and tables. Supplemental files are available at figshare. Figure S1 compares changes in pre-mRNA splicing identified using different software packages. Figure S2 provides a comparison of the current *in vivo* generated RNAseq data to a prior *in vitro* study (O’Connor *et al.* 2015). Figure S3 provides data about alternative splicing of Nemo and Cav1. Figure S4 provides data about alternative splicing of Idh3b and Idh3g. Figure S5 provides further data about measured metabolite levels in alveolar macrophages. Table S1 lists oligonucleotide sequences used in this study. Table S2 lists the results of DEXseq analysis to identify pre-mRNA splicing changes as outlined in the manuscript. Table S3 lists the results of DEXseq, MISO, and Cuffdiff analysis to identify pre-mRNA splicing changes as outlined in the manuscript. Table S4 lists pathway analyses of our gene expression and pre-mRNA splicing data. Table S5 lists the results of our metabolomics analysis. Gene expression data and sequence reads are available at the Gene Expression Omnibus (GEO) with the accession number GSE94749. Supplemental material available at figshare: https://doi.org/10.25387/g3.10565573.

## Results

### Intratracheal LPS administration induces a transient wave of inflammatory cytokine production

To characterize inflammatory readouts in our model of LPS-induced inflammation, we measured macrophage and neutrophil levels and pro-inflammatory cytokine concentrations in bronchoalveolar lavage (BAL). Neutrophil counts and pro-inflammatory cytokine levels were significantly elevated three days after LPS instillation (Figure 1A,C,E,G,I). Recruited alveolar macrophages (AMs) were present in the airspaces at this time point, with numbers similar to that of resident AMs (Figure 1B). By Day 6, pro-inflammatory cytokine levels had returned to baseline and the number of neutrophils was negligible (Figure 1A,C,E,G,I). Resident and recruited AM levels peaked at Day 6 and trended toward baseline by Day 12 (not shown). Accordingly, Day 3 represents a time point at which acute inflammation is present, whereas later time points represent the resolution of inflammation. To assess the potential contribution of resident *vs.* recruited AMs to the inflammatory milieu we interrogated our RNA-sequencing dataset (Mould *et al.* 2017). As we have shown previously, both resident and recruited AMs demonstrated significant expression of pro-inflammatory cytokines including TNFα, IL-6, IL-1β, and KC, at Day 3 (Mould *et al.* 2017), which was greater in recruited AMs than resident AMs (Figure 1D,F,H,J). By Day 6, pro-inflammatory cytokine gene expression, including these same four cytokines, returned to baseline in both macrophage subsets (Figure 1D,F,H,J).

### Pre-mRNA splicing is altered in alveolar macrophages during acute inflammation

**Figure 2:**
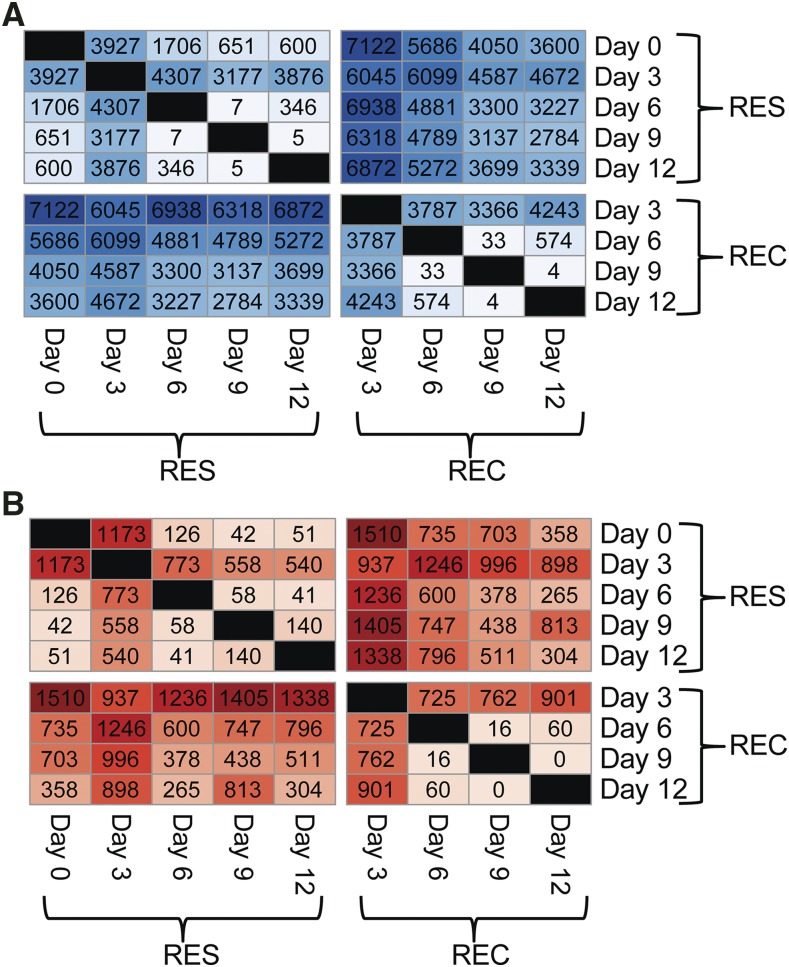
Inflammation induces transient alterations in gene expression and isoform usage in alveolar macrophages. Macrophages were isolated from naïve and LPS-treated mice by lung lavage. Resident and recruited alveolar macrophages were purified, RNA was prepared, and RNAseq was performed. Figures depict: (A) the number of significantly differentially expressed genes (results from pairwise DESeq2 comparisons, counting all genes with an adjusted p-value ≤ 0.05) or (B) the number of genes in which DEXSeq identified significant differences in at least one exon or exon fragment (after accounting for differential expression, adjusted p-value ≤ 0.05) for each pairwise comparison of sample groups. Color shades correspond to the magnitude of the numbers. Darker colors indicate comparisons with more differences (*i.e.*, more differential gene expression or isoform usage). RES refers to resident alveolar macrophages; REC refers to recruited alveolar macrophages. Day 0 refers to resident alveolar macrophages from untreated mice. There is no REC Day 0 sample, because these cells do not arrive until several days after LPS challenge.

We previously demonstrated that the transcriptional profiles of resident and recruited AMs vary markedly during inflammation (Mould *et al.* 2017). To quantify these changes in the context of alternative pre-mRNA splicing, we first performed pairwise comparisons of global gene expression between time points for each macrophage subset (Figure 2A). As a next step, we evaluated alternative pre-mRNA splicing using DEXSeq (Anders *et al.* 2012), assessed differential isoform usage (Table S2), and determined how many genes exhibited alternative isoform usage under the various pairwise comparisons (Figure 2B). In resident AMs, we identified over 1,000 genes with altered isoform usage on Day 3 *vs.* baseline (Day 0) and over 700 genes on Day 3 *vs.* Day 6. In comparison, only 126 genes were altered on Day 6 compared to baseline, and only 51 genes were altered on Day 12 compared to baseline. This suggests that in resident AMs, a transient wave of alternative pre-mRNA splicing occurs early after LPS challenge and that this wave of differential isoform usage resolves at later times. Likewise, alternate isoform usage was greatest in recruited AMs three days after challenge (Figure 2B), suggesting that recruited AMs also exhibit a largely transient alteration in pre-mRNA splicing. Note that recruited AMs are absent in the homeostatic lung and only exist during acute and resolving inflammation. In both resident and recruited AMs, the time-dependent changes in isoform usage paralleled changes in gene expression (compare Figure 2B to Figure 2A).

**Figure 3:**
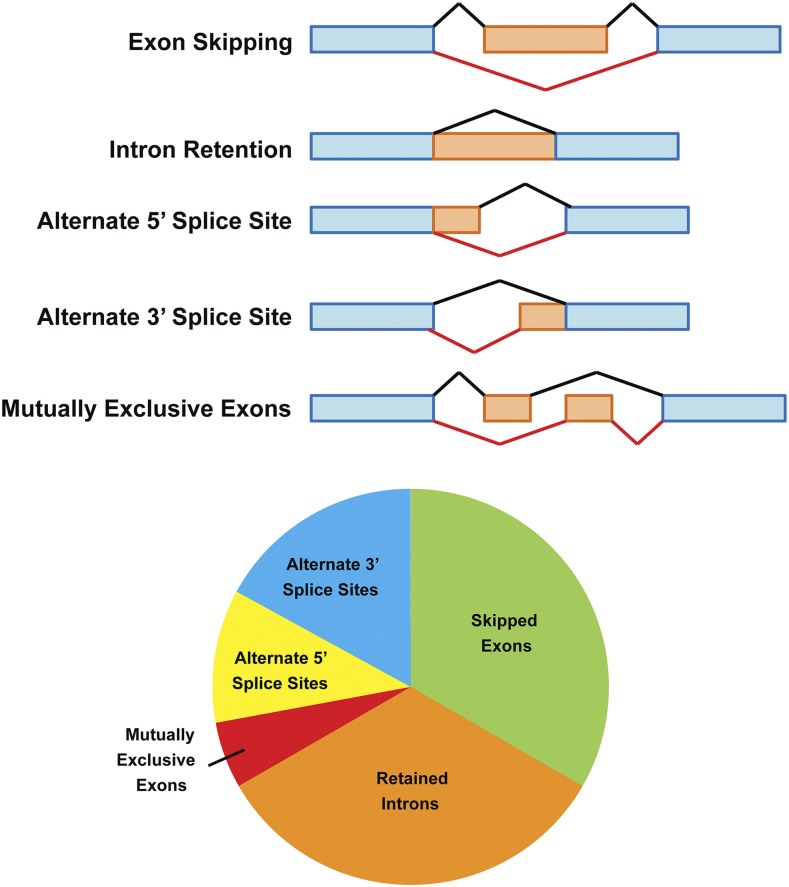
Inflammation induces multiple classes of alternative pre-mRNA splicing events in resident alveolar macrophages. MISO was used to identify alternative splicing in resident alveolar macrophages isolated from mice 3 days after LPS challenge compared to resident alveolar macrophages from untreated mice. Schematics at top depict the various classes of alternative splicing events. Black bars indicate the canonical splicing event; red bars and orange exons indicate the alternative pre-mRNA splicing event. The pie chart indicates the frequency of each class of alternative splicing event. Total N (number of alternative splicing events identified) = 276.

To further investigate the effect of inflammation on pre-mRNA splicing, we assessed isoform usage in resident AMs from each inflammatory time point and compared it to isoform usage by homeostatic AMs (Day 0). Three different software packages were used: DEXSeq, MISO, and Cuffdiff. As reported previously, different software packages identify overlapping but distinct sets of alternative splicing events in RNAseq data (Wu *et al.* 2018), and this was reflected in our analysis (Figure S1, Table S3). We next used MISO, which reports the nature of alternative splicing events, to classify specific forms of pre-mRNA alternative splicing induced by LPS (Figure 3 top). Since the greatest number of inflammation-induced splicing alterations in resident AMs occurred 3 days after LPS challenge, we chose to investigate this time point compared to untreated AMs (Day 0). The results demonstrate that one third of alternative splicing events resulted from skipped exons and one third represented retained introns (Figure 3 bottom). We also observed a smaller percentage of other classes of pre-mRNA splicing changes including mutually exclusive exon usage, altered 5′ splice site usage, and altered 3′ splice site usage. Thus, inflammation induces multiple classes of alternative pre-mRNA splicing events in resident AMs and does so in ratios similar to those reported for other signal-induced alternative splicing events (Stamm *et al.* 2012).

To determine what the potential effects of this alternative splicing might be, we analyzed the exon skipping events in resident AMs (Day 0 *vs.* 3) in more detail. Exons that are alternatively spliced are reported to be a multiple of three base pairs in length more often than expected by chance (>33%) (Barbosa-Morais *et al.* 2012; Magen and Ast 2005; Xing and Lee 2005), and this was reflected in our data. We found that 51% of inflammation-induced exon skipping events involved an exon that was a multiple of three base pairs in length. Thus, approximately half of the detected exon skipping changes are predicted to maintain the reading frame and are likely to either preserve some protein function or perhaps produce proteins of differing function. In contrast, approximately half of the detected exon skipping events introduce a frame-shift and are predicted to significantly perturb or even ablate protein function.

Finally, we assessed if LPS induced a similar set of alternative splicing events *in vivo* as it did *in vitro* by comparing our current *in vivo* results to our prior *in vitro* study in which we assessed alternative splicing induced by LPS in the mouse macrophage cell line RAW264.7 (O’Connor *et al.* 2015). For this analysis, we used DEXseq to identify alternative isoform usage and compared LPS-induced alternative splicing in resident AMs (Day 0 *vs.* 3) to LPS-induced alternative splicing in RAW264.7 cells (20 ng/ml LPS for 4 hr). There were substantially more alternative isoform events identified in the current *in vivo* study than in the prior *in vitro* study (Figure S2). This likely reflects the much longer timescale of the *in vivo* study and the inherent complexity of the *in vivo* milieu. However, despite the differences in the two model systems, there was substantial overlap in the genes that exhibited LPS-induced alternative isoform usage: 40 of 81 loci that exhibited altered isoform usage following *in vitro* LPS treatment also were identified following *in vivo* LPS treatment (Figure S2).

### Inflammation induces altered isoform usage in genes that modulate TLR signaling

We used qPCR to confirm the validity of several alternative pre-mRNA splicing events detected in the RNAseq analysis from our *in vivo* study. The response to LPS is mediated by Toll-like receptor 4 (TLR4) and its downstream signaling molecules (Kawai and Akira 2010; Takeuchi and Akira 2010). Therefore, we manually inspected isoform usage data (Table S3) for genes known to affect TLR signaling and genes that had the largest predicted changes in isoform usage. We chose to investigate two of these genes further: the NFκB regulatory protein *Nemo* (alias Ikbkg) (Ostuni *et al.* 2010) and *Caveolin 1* (Cav1) (Jiao *et al.* 2013; Wang *et al.* 2009; Garrean *et al.* 2006; Medina *et al.* 2006).

**Figure 4:**
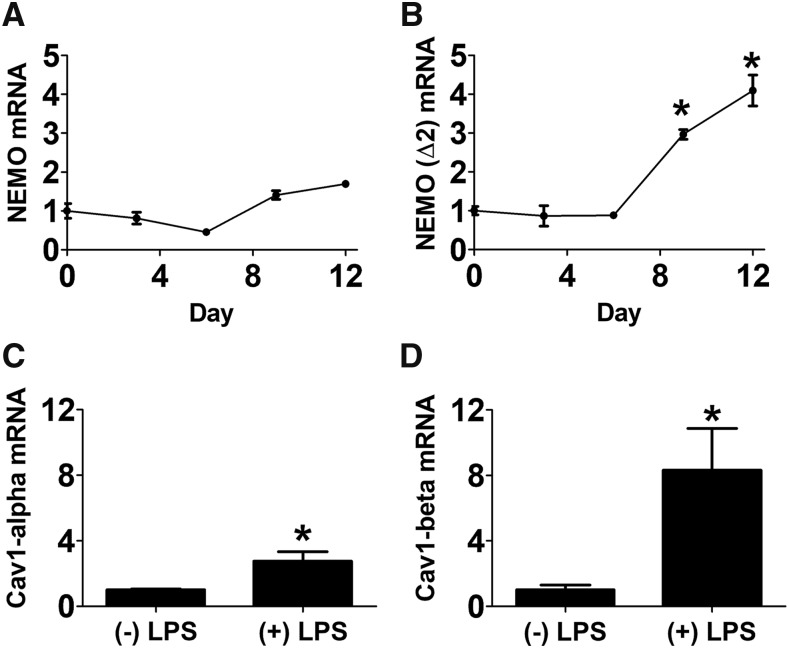
LPS induces altered isoform usage in genes that regulate Toll-like receptor signaling. The panels depict the results of qPCR assays used to monitor expression of the indicated mRNA isoforms in resident AMs. The data are normalized so that expression of each isoform in the absence of LPS is set to 1. The (+) LPS samples in panels C and D were collected 3 days after LPS challenge. Bars indicate mean, SEM. Asterisks indicate results that were significantly different from control (*P* < 0.05).

Nemo is part of the IKK complex required for activation of NFκB (Ostuni *et al.* 2010), and mutations that alter *Nemo* pre-mRNA splicing can cause severe immunodeficiency (Hanson *et al.* 2008; Maubach and Naumann 2017). Our RNAseq analysis identified a novel LPS-induced alternative splice form lacking exon 2 (NemoΔ2) that increased significantly at later times (Figure S3A). This splice form lacks the translation start site and therefore may produce non-functional mRNA. To verify the production of this novel Nemo isoform, we designed qPCR primers specific to wild type Nemo and NemoΔ2. Consistent with the RNAseq data, qPCR indicated that wild type Nemo mRNA levels remained relatively constant during inflammation (Figure 4A). In comparison, NemoΔ2 mRNA increased significantly at later times after LPS treatment (Figure 4B). Using RT-PCR with primers that bracket exon 2 and subsequent agarose gel electrophoresis to monitor both transcripts simultaneously, we confirmed that LPS induced NemoΔ2 mRNA (Figure S3B); however, NemoΔ2 mRNA represents only a minor fraction of the total Nemo mRNA pool (3.4 ± 2.1% in the absence of LPS, 8.3 ± 3.0% in the presence of LPS, mean± SEM, Figure S3B). Thus, the biological impact of up-regulation of this transcript is unclear.

Cav1, a component of caveolae, is a signaling molecule with multiple functions including modulation of TLR signaling (Jiao *et al.* 2013; Wang *et al.* 2009; Garrean *et al.* 2006; Medina *et al.* 2006). There are two known isoforms of *Cav1*, Cav1α and Cav1β. These two isoforms may have differential signaling functions and are formed by use of alternate transcription start sites (Fang *et al.* 2006; Fujimoto *et al.* 2000; Kogo and Fujimoto 2000). Our RNAseq analysis indicated that Cav1β mRNA levels were selectively induced in resident AMs 3 days after LPS challenge (Figure S3C). We designed isoform-specific primers and used qPCR to confirm these RNAseq data (Figure 4C,D). The qPCR data indicated that the Cav1α:Cav1β ratio in the absence of LPS was 9.9 ± 2.0:1 (mean± SEM); this ratio decreased to 2.7 ± 0.3:1 in the presence of LPS. These qPCR data (Figure 4C,D) are consistent with the RNAseq data (Figure S3C).

### Inflammation induces altered gene expression and altered pre-mRNA splicing in a common set of signaling pathways

We previously used pathway analysis to ascribe functional roles to the transcriptional profiles of resident and recruited AMs during inflammation (Mould *et al.* 2017). Our data showed that the largest differences occurred on LPS day 3, particularly in pathways associated with inflammation, cell proliferation and metabolism. To determine if these findings extended to pre-mRNA splicing, we cataloged genes with altered isoform usage in resident and recruited AMs from selected time points and used DAVID to interrogate pathways in the Kyoto Encyclopedia of Genes and Gene Products (KEGG) database. Specifically, we performed KEGG pathway analysis on genes IDs with altered isoform usage in the various comparisons. In parallel we performed KEGG pathway analysis on differentially expressed genes (DEGs), as done previously (Mould *et al.* 2017).

**Figure 5:**
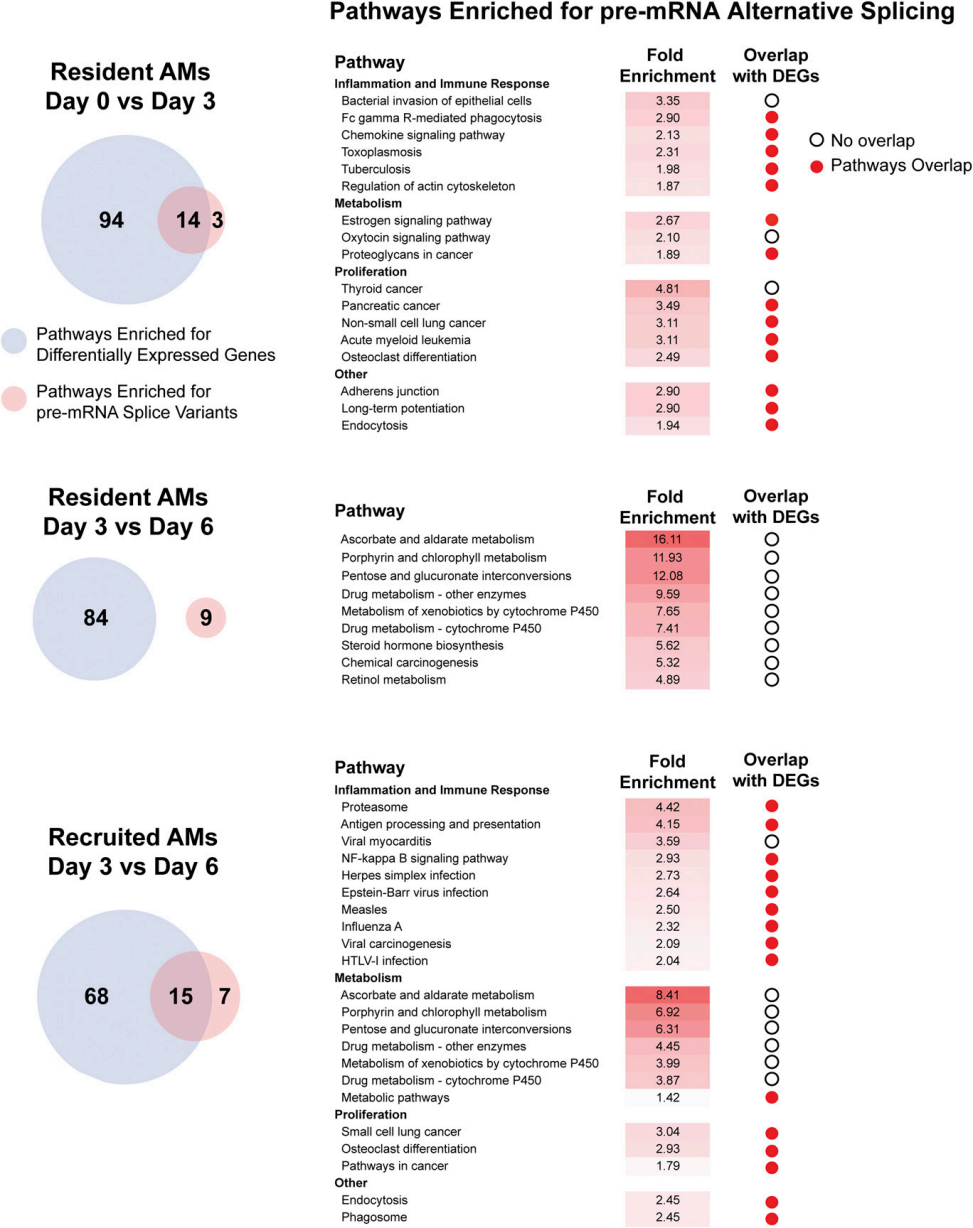
Acute inflammation induces differential gene expression and differential isoform usage in common sets of signaling pathways. Venn diagrams depict the number of KEGG pathways with significant enrichment of differentially expressed genes (DEGs) (light blue) and alternative pre-mRNA splice variants (pink). Pathways were determined by applying DAVID to assess DESeq2-derived gene expression changes or DEXSeq-derived pre-mRNA alternative splicing changes. Pathway analysis was performed on the unique gene IDs exhibiting any altered isoforms. Tables list pathways with significant enrichment of pre-mRNA splice variants. Major categories for pathways include inflammation and immune response, metabolism and cell proliferation. Red circles indicate pathways enriched for both DEGs and pre-mRNA splice variants. Open circles indicate pathways enriched for alternative splicing changes but not DEGs.

We first compared resident AMs isolated from naive mice and Day 3 LPS treated mice. Analysis of DEGs yielded 108 pathways, including multiple pathways associated with inflammation, cellular proliferation and metabolism (Figure 5, Table S4). In parallel, 17 pathways were enriched for mRNA splice variants. Of these, 14 (82%) overlapped with pathways enriched for DEGs. The main categories included inflammation and immune response, metabolism and cancer pathways that were highly enriched for proliferative genes. Next, resident AMs from LPS Day 3 and Day 6 were compared. 84 pathways were enriched for DEGs and nine were enriched for altered isoform usage; however, none of these pathways overlapped. Lastly, recruited AMs isolated 3 and 6 days after LPS treatment were compared. 83 pathways were enriched for DEGs, whereas 22 were enriched for alternative mRNA splice variants. Of these 15 overlapped. The main categories of overlapping pathways were inflammation and immune response, cell proliferation and metabolism. These results demonstrate that splicing changes induced during inflammation may target particular signaling pathways that are involved in modulating or mediating immune responses and cell metabolism. In this regard, it is likely that during acute inflammation, gene expression and pre-mRNA splicing are induced in common sets of signaling pathways and suggests that post-transcriptional regulation of the inflammatory response may contribute to macrophage function.

### Pre-mRNA splicing of metabolic pathway genes is altered in resident *vs.* recruited AMs

Cell metabolism is a critical regulator of macrophage activation and plays a key role in driving inflammatory outputs. In this context, pro-inflammatory macrophages have “Warburg-like” metabolism, characterized by enhanced flux through glycolysis and decreased flux through the TCA cycle (Jha *et al.* 2015; Rodríguez-Prados *et al.* 2010). We have previously shown that 3 days after LPS challenge, recruited AMs are more pro-inflammatory than resident AMs and that they have greater expression of glycolytic enzyme genes and decreased expression of TCA cycle genes (Mould *et al.* 2017). This metabolic difference was also reflected at the isoform usage level, as all four KEGG pathways with significantly differential isoform usage between day 3 resident and recruited AMs were metabolic pathways (Table S4). Likewise, all eight KEGG pathways with significantly altered isoform usage between day 6 resident and recruited AMs were metabolic pathways (Table S4).

**Figure 6:**
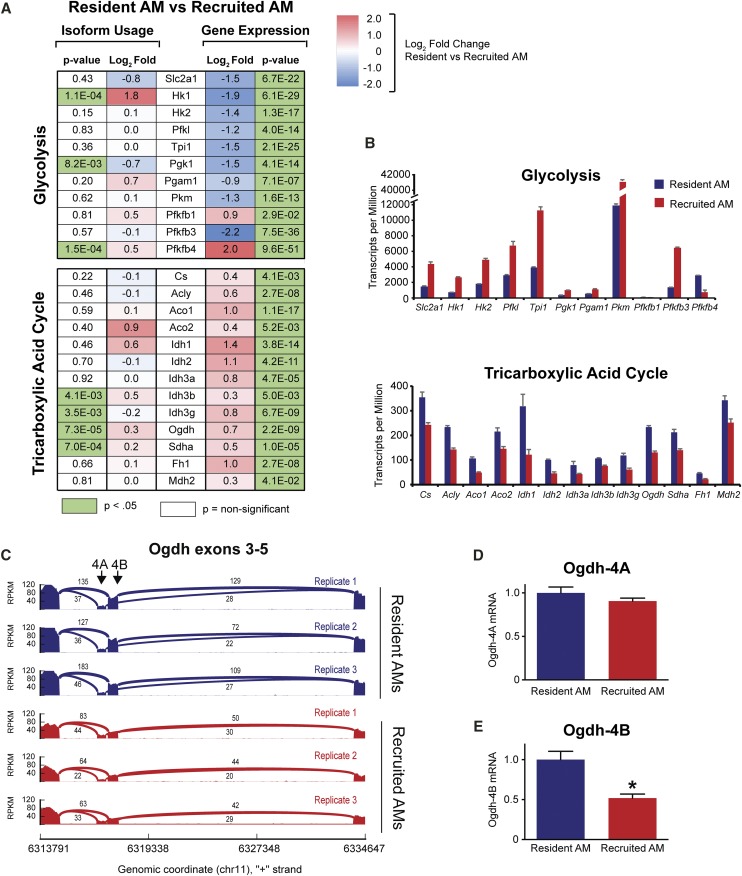
Differential gene expression and isoform usage in metabolic pathways for resident *vs.* recruited alveolar macrophages. Mice were treated with 20 μg LPS instilled into the lungs. Resident and recruited AMs were isolated from bronchoalveolar lavage 3 days later and were purified using FACS. (A) Isoform usage determined with DEXSeq and differential gene expression for components of the glycolytic pathway and tricarboxylic acid cycle. Log_2_ fold-change of pre-mRNA splice variants and gene expression is shown. p-values are adjusted for multiple testing. (B) Transcripts per million of glycolytic and tricarboxylic acid cycle genes from RNA-seq analysis. (C) Sashimi plots (Katz *et al.* 2010) depict altered usage of exons 4A and 4B in Ogdh resident and recruited AMs. The peaks show the read coverage as reads per kilobase and million mapped reads (RPKM), which represents the relative expression of each exon. The numbers above the junction-spanning links and their line widths indicate the number of junction-spanning reads. (D,E) qPCR was used to measure expression of the indicated mRNA isoforms using isoform-specific primers as outlined in the Material and Methods; data are normalized relative to the housekeeping gene βactin using the ddCt method. Data are normalized so that expression of each isoform in resident alveolar macrophages is set to 1. N = 3 biologic replicates per group. Bars indicate mean, SEM. *P < 0.05. Glycolysis enzymes: *Slc2a1* = solute carrier family 2, facilitated glucose transporter (also known as GLUT1), *Hk1* = hexokinase 1, *Hk2* = hexokinase 2, *Pfkl* = phosphofructokinase, *Tpi1* = triose phosphate isomerase, *Pgam1* = phosphoglyceratemutase 1, *Pkm*= pyruvate kinase, *Pfkfb* = 6-phosphofructo-2-kinase/fructose-2,6-biphosphatase. Tricarboxylic acid cycle components: *Cs* = citrate synthase, *Acly* = ATP citrate lyase, *Aco* = aconitase, *Idh* =isocitrate dehydrogenase, Ogdh = oxoglutarate dehydrogenase (also known as α-ketoglutarate dehydrogenase), *Sdh* = succinate dehydrogenase, *Fh1* = fumarate hydratase 1, *Mdh2* = malate dehydrogenase.

To investigate how altered pre-mRNA splicing impacts these metabolic pathways, we interrogated our gene expression and alternative pre-mRNA splicing datasets in parallel. We found that 11 genes of the glycolytic pathway were more highly expressed by recruited AMs, whereas 13 genes encoding TCA cycle components were more highly expressed in resident AMs (Figure 6A, B). Of these, pre-mRNA splicing was significantly altered in three glycolytic and four TCA cycle genes. We decided to explore the four TCA cycle genes with altered splicing further: *Idh3g* and *Idh3b* (two of three members of the isocitrate dehydrogenase 3 complex), *Ogdh* (*oxoglutarate dehydrogenase*), and *Sdha* (succinate dehydrogenase complex flavoprotein subunit A).

The alternative pre-mRNA splicing event in *Idh3b* resulted in increased retention of intron 4 in recruited AMs (Figure S4A). The canonical isoform of *Idh3b* encodes a 384 amino acid protein that contains the complete Isocitrate/isopropylmalate dehydrogenase domain (amino acids 46-379). The alternate isoform that retains intron 4 introduces a premature stop codon; thus, this isoform encodes a truncated 144 amino acid protein that includes 112 canonical amino acids and 32 variant amino acids. This transcript variant lacks most of the conserved Isocitrate/isopropylmalate dehydrogenase domain and is predicted to weaken Idh3 function. Moreover, it is possible that this transcript will be subject to nonsense-mediated decay (NMD) (Hug *et al.* 2016). Idh3g had a moderate decrease in the inclusion of the 3′ end of exon 5 in recruited AMs (Figure S4B); it is unclear what effect this might have, but we speculate that it might also reduce Idh3 function. Together, these pre-mRNA splice variants are expected to reduce activity of the isocitrate dehydrogenase complex. Sdha exhibited a small but significant increase in 3′ UTR levels in recruited AMs compared to resident AMs, which we speculate could modify Sdha mRNA mRNA localization, mRNA stability, or translation (Zhao *et al.* 2011; Matoulkova *et al.* 2012; Mayr 2019).

In addition to an overall decrease in expression in recruited AM (Figure 6A,B), Ogdh exhibited altered isoform usage between the two classes of AMs (Figure 6C). The alternative pre-mRNA splicing in *Ogdh* resulted in the inclusion of either of two mutually exclusive exons, which we have termed exons 4A and 4B (Figure 6C). The canonical Ogdh isoform includes exon 4B (Ogdh-4B), and analysis of our RNAseq data suggests that this isoform represents almost all of the mRNA in resident AMs (Figure 6C). In comparison, the Ogdh-4A to Ogdh-4B mRNA ratio was significantly increased in recruited AMs, with similar levels of the two splice forms present (Figure 6C). To confirm these findings, we used isoform-specific primers and qPCR, which demonstrated a significant decrease in Ogdh-4B but no significant change in Ogdh-4A mRNA in recruited AMs compared to resident AMs (Figure 6D, E). This resulted in a decreased ratio of Ogdh-4B to Ogdh-4A mRNA in recruited AMs (2.0 ± 0.2:1 mean± SEM) compared to resident AMs (3.4 ± 0.3:1). We speculate that reduced expression of the canonical splice form of *Ogdh* in recruited AMs leads to decreased Ogdh activity in recruited AMs. Consistent with this speculation, the Ogdh-4A splice form deletes a Ca^2+^ binding site that is required for Ca^2+^-induced activation of Ogdh activity (Denton 2009; Armstrong *et al.* 2014; Denton *et al.* 2016). This splice form also is increased during metabolic shifts associated with neuronal differentiation (Zheng *et al.* 2016).

Taken as a whole, the gene expression and pre-mRNA splicing changes identified in TCA cycle genes are predicted to decrease the activity of this pathway in recruited AMs.

### Metabolic flux through the TCA cycle is reduced in recruited AMs compared to resident AMs during acute inflammation

**Figure 7:**
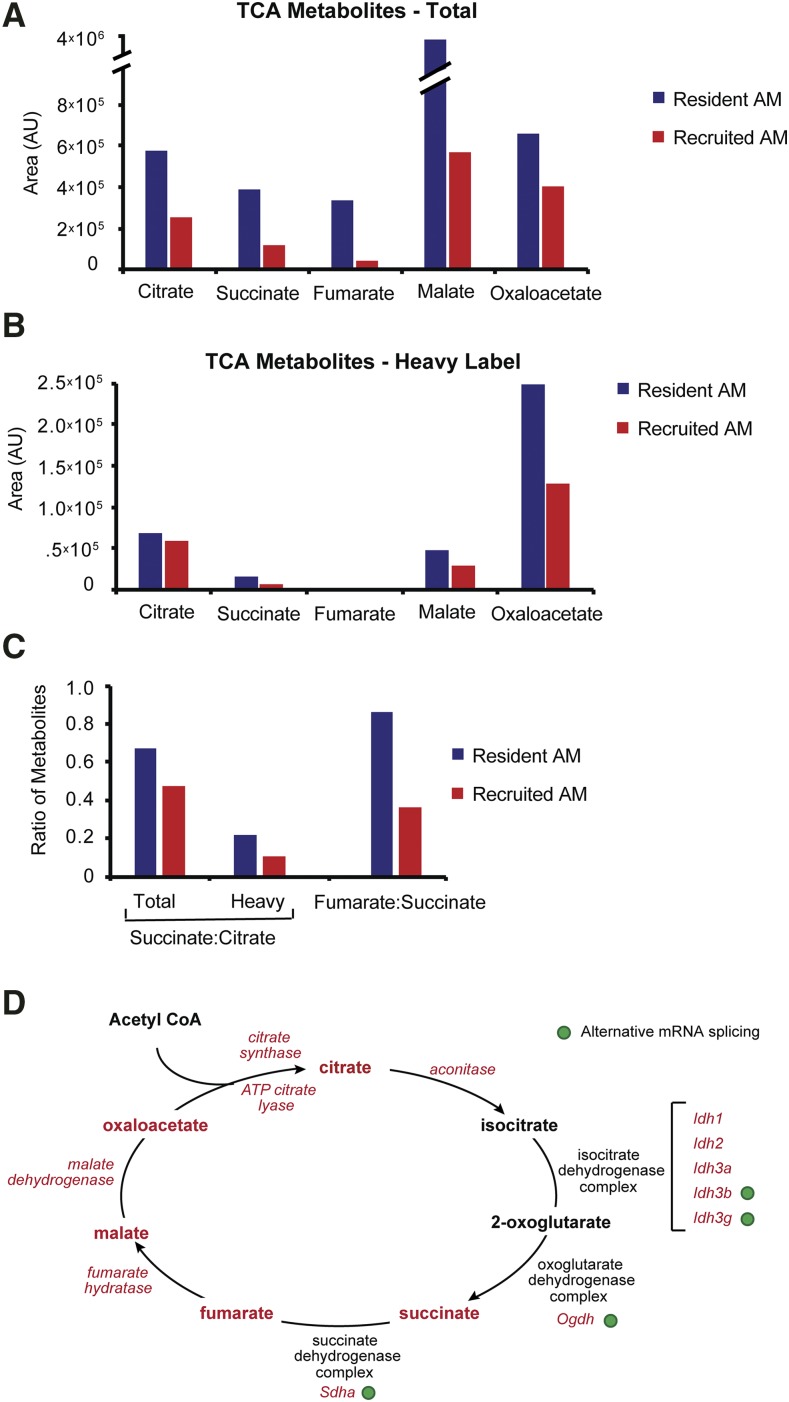
Recruited alveolar macrophages have decreased flux through the tricarboxylic acid cycle. U-^13^C-glucose (“heavy label”) was intravenously injected into mice on LPS Day 3 at 30 and 60 min prior to euthanasia. Resident and recruited AMs were purified by fluorescence activated cell sorting. (A, B) Total and isotope labeled TCA metabolites were measured with ultra-high-performance liquid chromatography / mass spectrometry. (C) Calculated metabolite ratios. (D) Schematic depicting integration of metabolomic, gene expression, and alternative pre-mRNA splicing data for the tricarboxylic acid cycle in resident compared to recruited AMs at day 3. Metabolites increased in resident AMs are indicated in red. Isocitrate and 2-oxoglutarate levels were not detectable at meaningful levels. Genes significantly downregulated in recruited AMs are shown in red italics. Green circles indicate genes with significantly altered isoform usage (adjusted *P* < 0.05).

To determine if the alterations in gene expression and mRNA isoform usage that we identified in TCA cycle components were associated with reduced metabolic flux through the pathway in recruited AMs, we intravenously administered U-^13^C-glucose to LPS-treated mice *in vivo* and performed targeted metabolomics on freshly isolated resident and recruited AMs three days after intratracheal LPS instillation (Table S5). Lactate and the lactate:pyruvate ratio were increased in recruited AMs as were levels of ^13^C-glucose 6-phosphate and ^13^C-pyruvate (Figure S5A-C). Moreover, in recruited AMs the fractions of glucose-6-phosphate, fructose 1,6-bisphosphonate, and pyruvate that contained the heavy-label were also increased (Figure S5D). Taken as a whole these data demonstrate enhanced glycolytic flux in recruited AMs compared to resident AMs, although the unmeasured efflux of ^13^C-lactate from the cell serves as a potential confounder for this analysis. In comparison, levels of both total and ^13^C-labeled TCA metabolites were reduced in recruited AMs compared to resident AMs (Figure 7A, B). To further confirm that changes in gene expression and pre-mRNA alternative splicing were associated with reduced activity of the isocitrate dehydrogenase and oxoglutarate dehydrogenase complexes in recruited AMs, we assessed ratios of succinate:citrate for total and heavy-labeled metabolites. As anticipated, these were reduced in recruited AMs compared to resident AMs (Figure 7C). A similar reduction in the fumarate:succinate ratio was observed, suggesting decreased activity of the succinate dehydrogenase complex. No differences were observed between resident and recruited AMs for fumarate:malate and malate:oxaloacetate (data not shown). Combined genetic and metabolic data are summarized in (Figure 7D).

## Discussion

Macrophages exposed to inflammatory stimuli undergo complex alterations in gene expression that regulate their biological response (Medzhitov and Horng 2009; Takeuchi and Akira 2010). These alterations in gene expression have been extensively studied both on a gene-by-gene basis and genome-wide using microarrays and more recently RNAseq. While these techniques have provided extraordinary insights into the mechanisms that regulate macrophage activation, they only tell part of the story since more than 95% of human genes produce more than one protein product. Accordingly, alternative pre-mRNA splicing plays a major role in shaping the proteome both during health and disease. In the context of acute inflammation, *in vitro* studies have shown that changes in alternative splicing can regulate both the amplitude and duration of inflammation (Lynch 2004; Schaub and Glasmacher 2017). While many of these alternative splicing events have been identified and studied on a gene-by-gene basis, there have been few genome-wide studies examining inflammation-associated alternative pre-mRNA splicing, and most, if not all, of these studies have been performed *in vitro*.

In the current study, we leveraged our previously published RNA sequencing data set (Mould *et al.* 2017) to examine alternative pre-mRNA splicing in resident and recruited AMs during acute and resolving inflammation *in vivo*. Our data demonstrate a high prevalence of alternative pre-mRNA splicing in both AM subsets and show that these encompassed multiple classes of alternative splicing events. Because approximately one-third of these were intron retention events, it is possible that many of the resulting mRNAs are subject to nonsense-mediated mRNA decay (NMD) (Hug *et al.* 2016).

Importantly, our analysis also identified strong temporal relationships. Alternative pre-mRNA splicing was maximal 3 days after LPS challenge and returned toward baseline levels at later times. Changes in global gene expression followed a similar kinetic profile and were the greatest 3 days after challenge, a time when the inflammatory response (as assayed by cytokine production and inflammatory cell accumulation in the lung) is at its peak. As inflammation resolved, changes in gene expression and alternative pre-mRNA splicing became less frequent. Our analysis further demonstrated that changes in pre-mRNA splicing and gene expression occurred in common sets of signaling pathways. Notably, these included pathways that regulate immune responses and cell metabolism.

Cell metabolism serves as a critical regulator of macrophage activation. In this regard, stimulation of macrophages with LPS *in vitro* leads to Warburg-like metabolism with enhanced glycolysis and inhibition of the TCA cycle (Jha *et al.* 2015; Rodríguez-Prados *et al.* 2010). Our previous work suggested that similar metabolic reprogramming occurred in AMs during the peak of acute inflammation, especially in recruited AMs (Mould *et al.* 2017). In the current study we verified these findings using targeted metabolomics. In addition, pulse-chase studies with U-^13^C-glucose revealed breaks in the TCA cycle that prevented metabolism of citrate to succinate and succinate to fumarate in recruited AMs. In a properly functioning TCA cycle, citrate is metabolized to succinate in three steps with isocitrate and 2-oxoglutarate as intermediates (see Figure 7D). Unfortunately, the concentrations of these intermediates were below the level of detection of our assays, preventing identification of the exact breakpoint. Notably, gene expression of *aconitase*, *Idh* and *Ogdh* were each reduced in recruited AMs. At the same time, alternative pre-mRNA splicing was identified in components of both the IDH and Ogdh complexes. The Ogdh isoform produced in recruited AM should exhibit decreased enzymatic activity (Denton 2009; Armstrong *et al.* 2014; Denton *et al.* 2016). Likewise, the Idh3b alternate isoform will truncate most of the protein, likely also resulting in reduced enzymatic function. For Idh3g, we speculate that the effect of alternative pre-mRNA splicing was reduced enzymatic activity. We postulate that flux from citrate to succinate was reduced at each metabolic step and that alterations in gene expression and alternative pre-mRNA splicing both contributed. However, since gene expression and alternative pre-mRNA splicing changes were directionally concordant, we are unable to determine which had a greater biologic effect.

Our study has several limitations. First, different software packages have been reported to identify overlapping but distinct sets of alternative splicing events in RNAseq data (Wu *et al.* 2018). Accordingly, while analysis of our RNAseq may not comprehensively identify all alternative pre-mRNA splicing events, it most likely captures a representative sample of most such events. Second, unlike analysis of gene expression in which one can infer the likely effect of expression changes (*i.e.*, increased or decreased), the effect of altered pre-mRNA splicing is not always readily apparent, and the cumulative effect of splicing changes on signaling pathways has to be inferred. Moreover, the observed magnitude of the changes in pre-mRNA splicing were often smaller than the changes in gene expression, further complicating our ability to differentiate the contribution of altered splicing and altered gene expression to commonly affected signaling pathways. Third, splicing alterations were most prevalent at the earliest time point examined (day 3); it would be interesting in future studies to investigate earlier time points to see if the extent of altered splicing is magnified soon after inflammation initiates.

In summary, our data demonstrate that inflammation induces substantial alternative pre-mRNA splicing in both resident and recruited AMs and that multiple classes of alternative pre-mRNA splicing events are encompassed. Moreover, these alternative splicing changes are greatest at the peak of inflammation and involve pathways that govern immune responses and metabolism. Notably, many of the pathways that are targeted by alternative splicing are shared with pathways enriched by differentially expressed genes, suggesting that these alternate splicing events play an important role in modulating the biological response to inflammation. In this context, our data show that alternative pre-mRNA splicing affects Cav1 and Nemo, key members of the TLR4 signaling pathway. Similarly, our data show that alternative pre-mRNA splicing of TCA cycle members is associated with reduced metabolic flux through this pathway. Together, these findings elucidate potential mechanisms by which alternative pre-mRNA splicing may alter inflammatory and metabolic programming of macrophages during acute and resolving inflammation.
